# Immunotherapy with VGX-3100 (HPV16 and HPV18 plasmids) + INO-9012 (DNA encoding IL-12) in human papillomavirus (HPV) associated head and neck squamous cell carcinoma (HNSCCa): interim safety and immunogenicity results

**DOI:** 10.1186/2051-1426-3-S2-P426

**Published:** 2015-11-04

**Authors:** Charu Aggarwal, Roger Cohen, Matthew P Morrow, Joshua Bauml, Gregory Weinstein, Jean Boyer, Xuefei Shen, Jian Yan, Jessica Goldenberg, Drishty Nashit, Sandra Oyola, Jessica Lee, Laurent M Humeau, David B Weiner, Zane Yang, Mark L Bagarazzi, David Weiner

**Affiliations:** 1 University of Pennsylvania, Philadelphia, PA, USA; 2 Inovio Pharmaceuticals, Plymouth Meeting, PA, USA; 3 Inovio Pharmaceuticals, San Diego, CA, USA; 4 Inovio, Philadelphia, PA, USA; 5 Inovio Pharmaceuticals, Inc., Plymouth Meeting, PA, USA

## Background

Oropharyngeal HNSCCa is frequently associated with HPV infection. DNA-based Immunotherapy with plasmids encoding HPV16 and HPV18 E6/E7 antigens has been shown to generate robust immune responses in women with HPV-driven high-grade cervical dysplasia. We hypothesize that HPV-specific immunotherapy with INO-3112 (VGX-3100 + INO-9012) in patients with HPV-associated HNSCCa will generate robust immunity which may contribute to disease stabilization or regression.

## Method

Eligibility for this prospective Phase I/IIa trial included adults with HPV-positive (assessed by p16) HNSCCa, ECOG PS 0-1, and adequate organ function. Patients (pts) are enrolled into two cohorts. In Cohort 1, pts receive INO-3112 pre and post-surgery. In Cohort 2, pts receive INO-3112 after completion of cisplatin based chemoradiation. INO-3112 (6mg of VGX-3100 plus 1mg of INO-9012) is delivered IM followed by electroporation with the CELLECTRA® device, once every 3 weeks for a total of 4 doses. Pts are followed for 2 years. Primary and secondary endpoints are safety and immune responses. Exploratory endpoints include: anti-tumor effect and progression-free-survival. Assessment of post-immunotherapy surgical specimens is being done to evaluate vaccine-induced lymphocyte infiltration in tumor.

## Results

As of June 2015, 19 pts have been enrolled. Complete safety data is available for 13 pts. Cohort 1: n=3, Cohort 2: n=10; 12 males; median age 57.7 years (range 39-76); cancers at base of tongue=6, tonsil=6, soft palate=1; never smoker=5, median follow-up is 104 days. INO-3112 was well tolerated with no Grade 3 or higher AEs. The most common AEs were injection site pain (n=11), local erythema (n=4) and hematoma/swelling (n=2, each). Two subjects had Grade 3 lymphopenia at baseline and no worsening during the trial. There was a Grade 2, unrelated SAE of post-surgical procedure hemorrhage. Enrollment and correlative analysis are ongoing; among samples tested to date, as compared to baseline, 4 of 5 evaluable pts showed elevated anti HPV16 and 18 E6/E7 antibody titers. Nine of 10 evaluable pts exhibited increased HPV-specific cellular responses by IFN-gamma ELISpot. Seven of 8 evaluable pts had HPV-specific CD8+ T cell activation concurrent with increased lytic proteins (granzymes and perforin) by flow cytometric analysis.

## Conclusion

These interim results demonstrate that this DNA-based immunotherapy (INO-3112) can safely generate HPV-specific CD8 T cell immunity in patients with HPV-related HNSSCa. All tested pts had positive cellular immune responses in at least one assay.

This study (NCT02163057) is co-sponsored by Inovio and the Abramson Cancer Center at the University of Pennsylvania (5P30CA016520-39).

## Trial Registration

ClinicalTrials.gov identifier NCT02163057.

